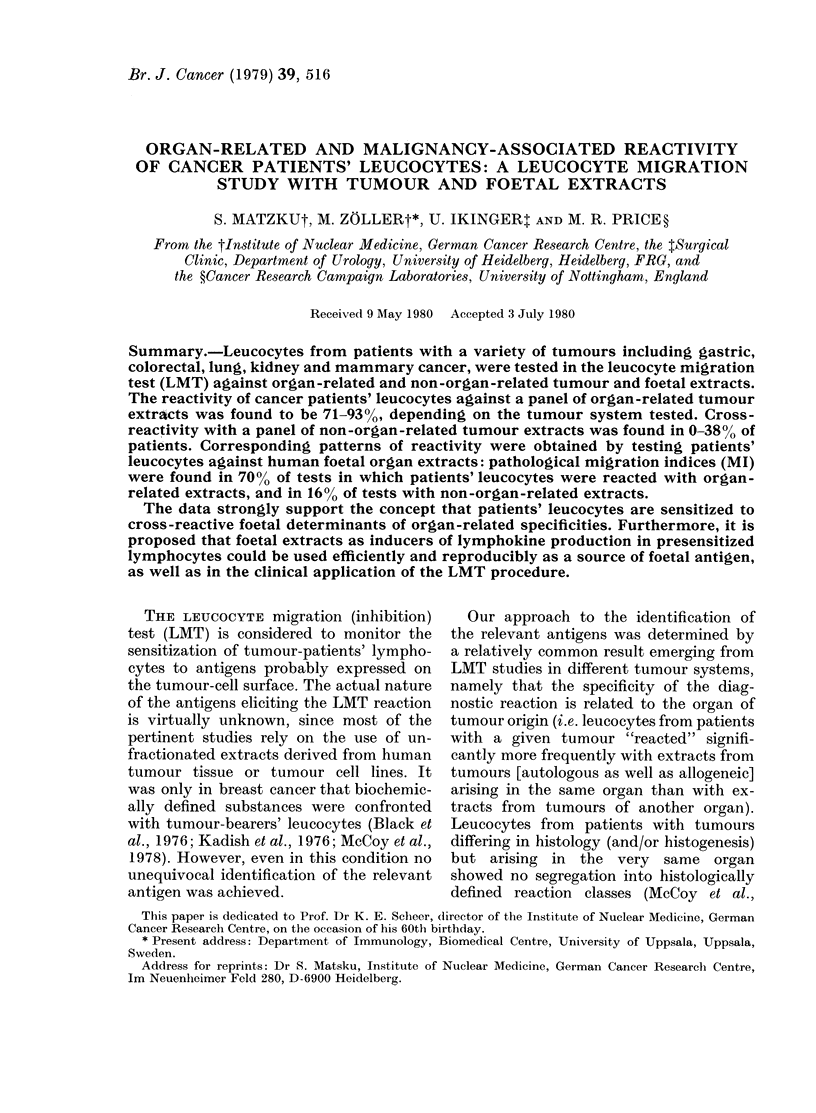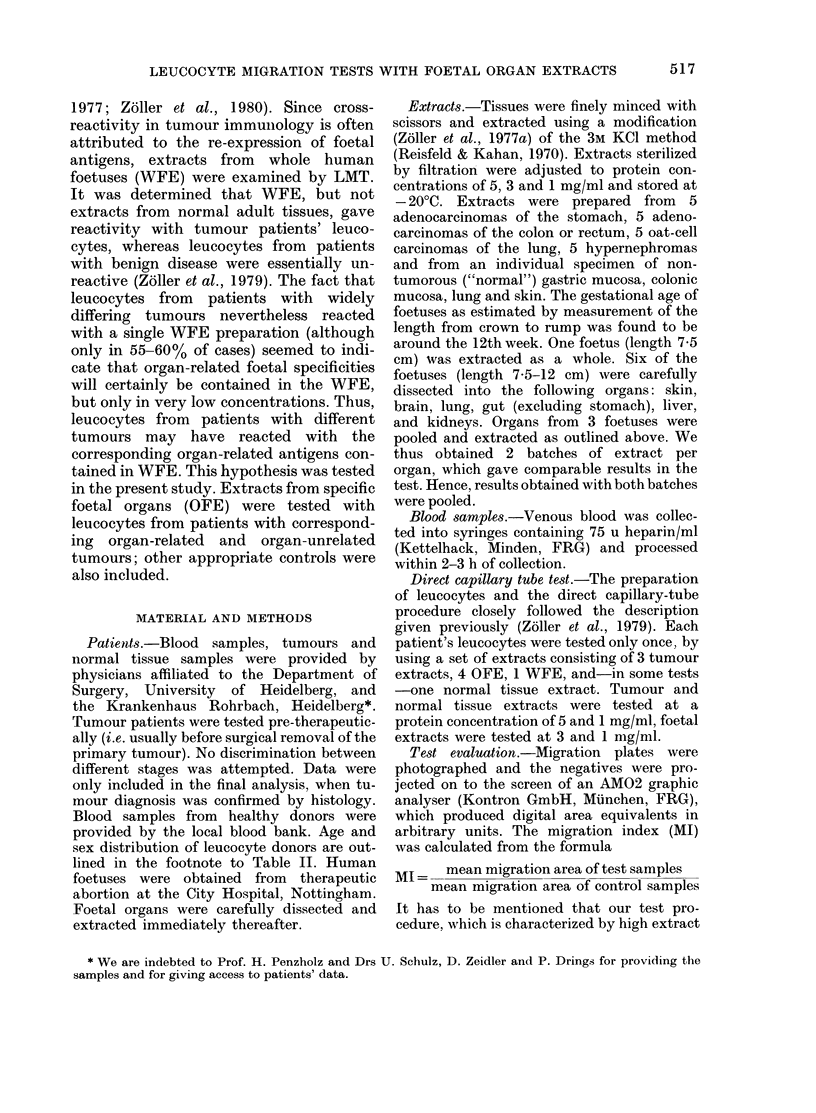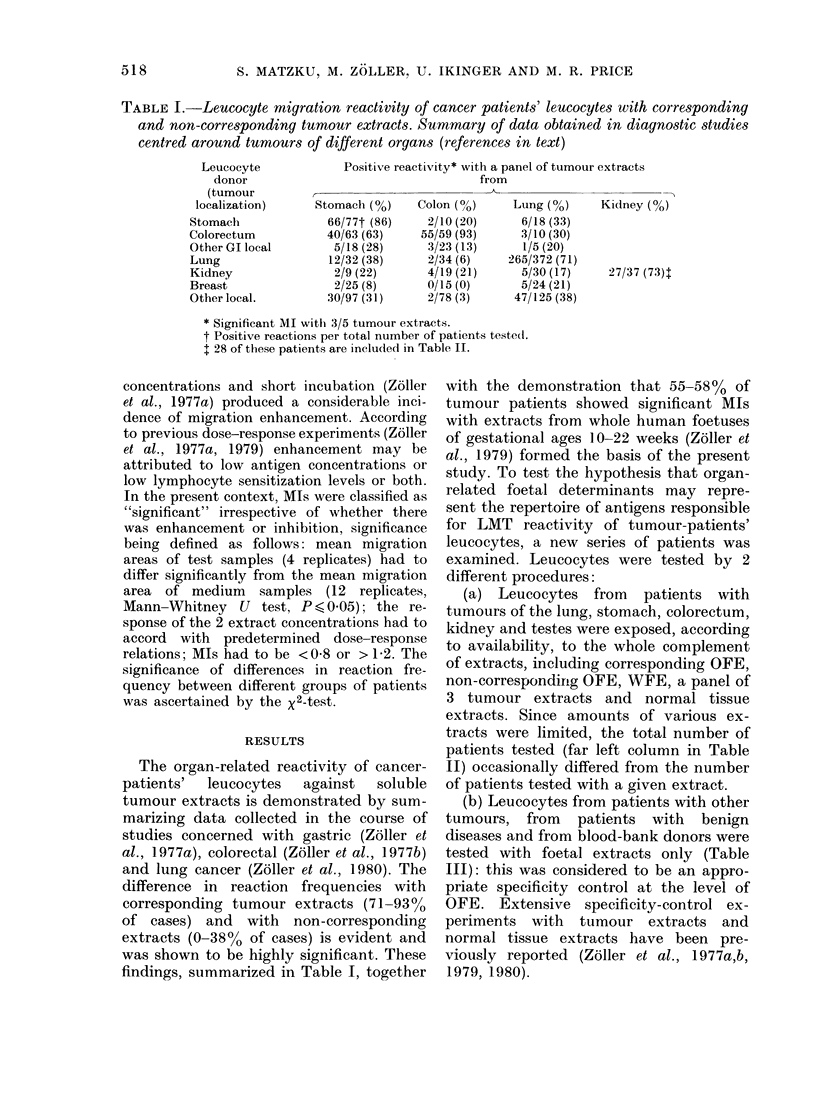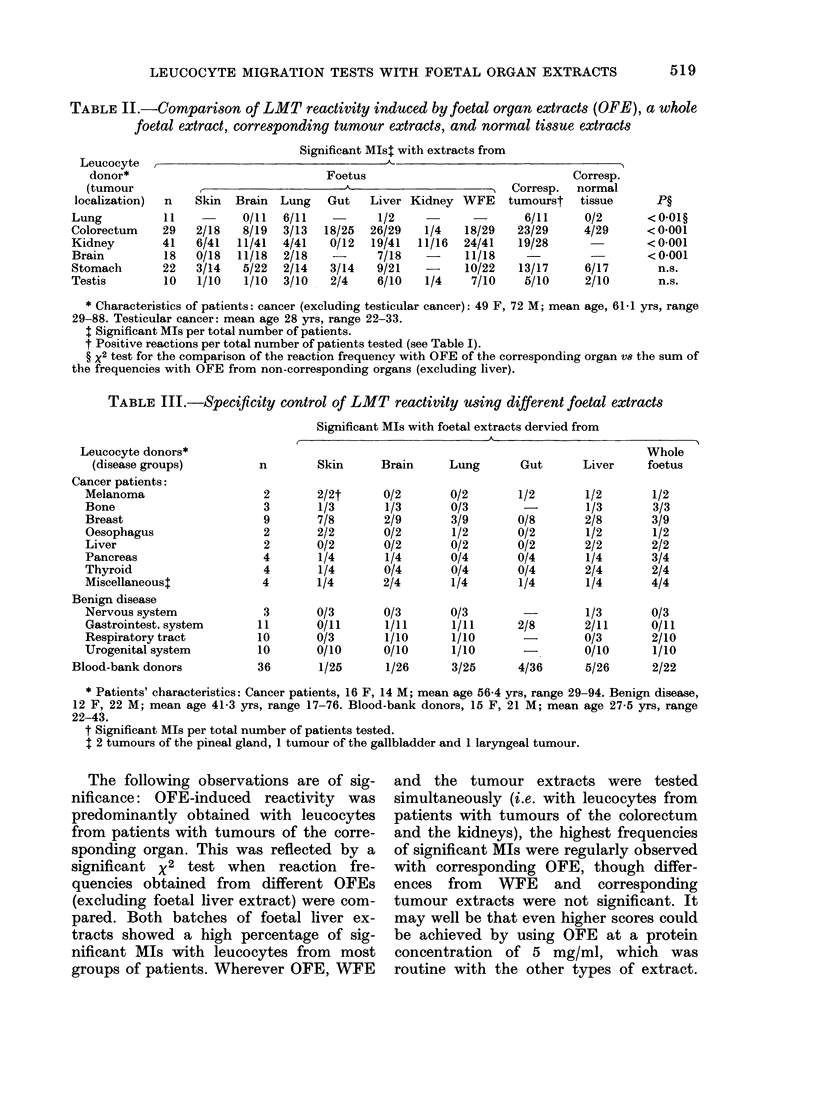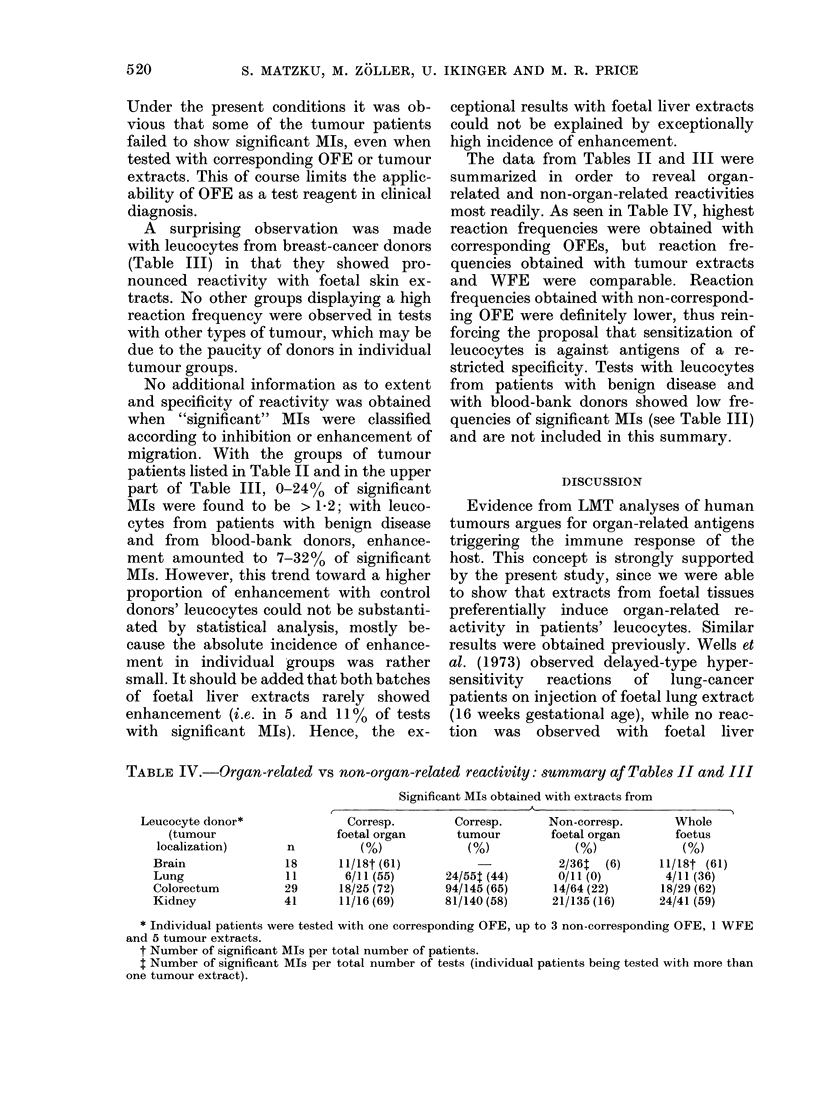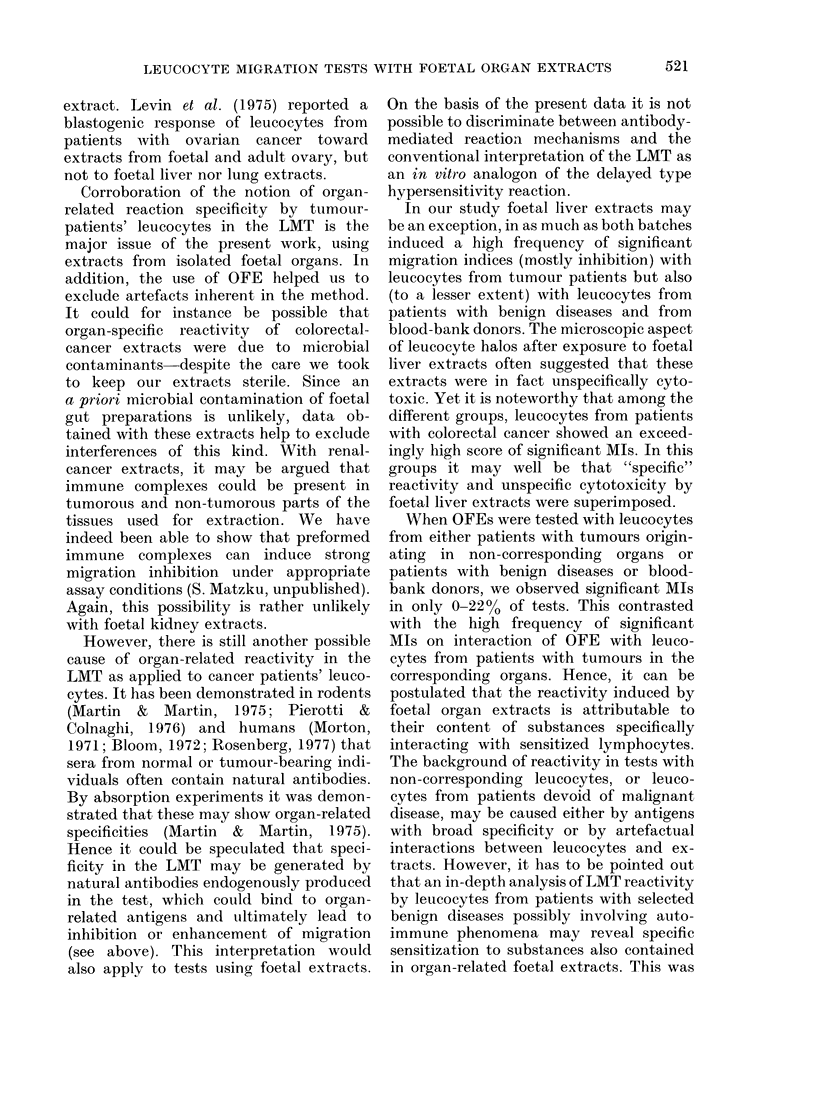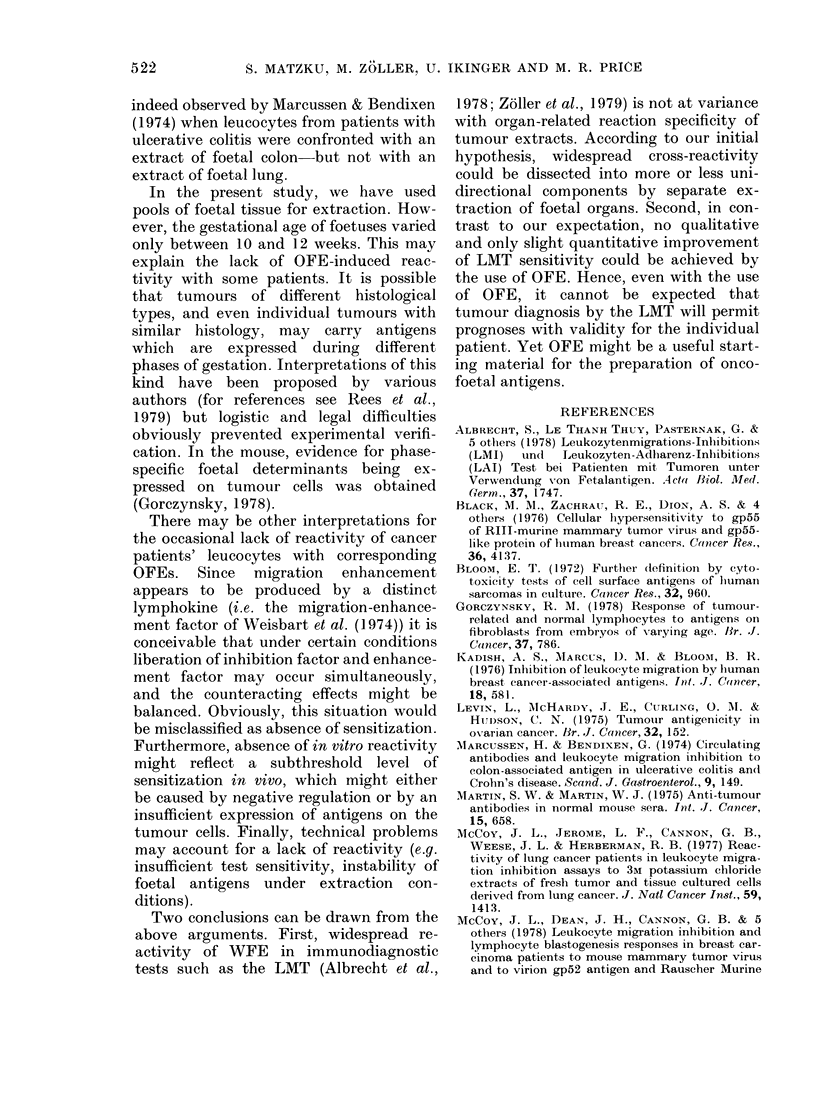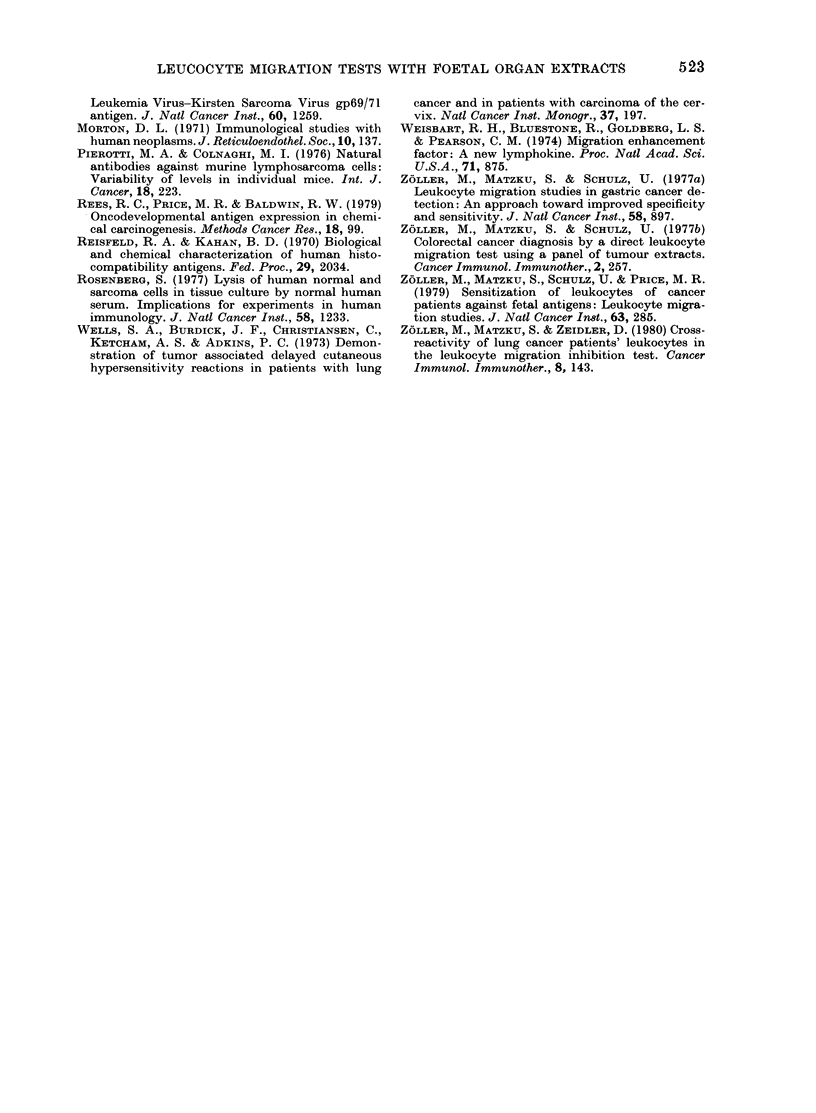# Organ-related and malignancy-associated reactivity of cancer patients' leucocytes: a leucocyte migration study with tumour and foetal extracts.

**DOI:** 10.1038/bjc.1980.274

**Published:** 1980-10

**Authors:** S. Matzku, M. Zöller, U. Ikinger, M. R. Price

## Abstract

Leucocytes from patients with a variety of tumours including gastric, colorectal, lung, kidney and mammary cancer, were tested in the leucocyte migration test (LMT) against organ-related and non-organ-related tumour and foetal extracts. The reactivity of cancer patients' leucocytes against a panel of organ-related tumour extracts was found to be 71-93%, depending on the tumour system tested. Cross-reactivity with a panel on non-organ-related tumour extracts was found in 0-38% of patients. Corresponding patterns of reactivity were obtained by testing patients' leucocytes against human foetal organ extracts; pathological migration indices (MI) were found in 70% of tests in which patients' leucocytes were reacted with organ-related extracts, and in 16% of tests with non-organ-related extracts. The data strongly support the concept that patients' leucocytes are sensitized to cross-reactive foetal determinants of organ-related specificities. Furthermore, it is proposed that foetal extracts as inducers of lymphokine production in presensitized lymphocytes could be used efficiently and reproducibly as a source of foetal antigen, as well as in the clinical application of the LMT procedure.


*Br. J. Cancer* (1979) **39**, 516

### ORGAN-RELATED AND MALIGNANCY-ASSOCIATED REACTIVITY OF CANCER PATIENTS' LEUCOCYTES: A LEUCOCYTE MIGRATION STUDY WITH TUMOUR AND FOETAL EXTRACTS

S. MATZKU†, M. ZÖLLER†*, U. IKINGER‡ AND M. R. PRICE§

*From the †Institute of Nuclear Medicine, German Cancer Research Centre, the ‡Surgical Clinic, Department of Urology, University of Heidelberg, Heidelberg, FRG, and the §Cancer Research Campaign Laboratories, University of Nottingham, England*

Received 9 May 1980 Accepted 3 July 1980

**Summary.—Leucocytes from patients with a variety of tumours including gastric, colorectal, lung, kidney and mammary cancer, were tested in the leucocyte migration test (LMT) against organ-related and non-organ-related tumour and foetal extracts. The reactivity of cancer patients' leucocytes against a panel of organ-related tumour extracts was found to be 71–93%, depending on the tumour system tested. Cross-reactivity with a panel of non-organ-related tumour extracts was found in 0–38% of patients. Corresponding patterns of reactivity were obtained by testing patients' leucocytes against human foetal organ extracts: pathological migration indices (MI) were found in 70% of tests in which patients' leucocytes were reacted with organ-related extracts, and in 16% of tests with non-organ-related extracts.**

**The data strongly support the concept that patients' leucocytes are sensitized to cross-reactive foetal determinants of organ-related specificities. Furthermore, it is proposed that foetal extracts as inducers of lymphokine production in presensitized lymphocytes could be used efficiently and reproducibly as a source of foetal antigen, as well as in the clinical application of the LMT procedure.**

THE LEUCOCYTE migration (inhibition) test (LMT) is considered to monitor the sensitization of tumour-patients' lymphocytes to antigens probably expressed on the tumour-cell surface. The actual nature of the antigens eliciting the LMT reaction is virtually unknown, since most of the pertinent studies rely on the use of unfractionated extracts derived from human tumour tissue or tumour cell lines. It was only in breast cancer that biochemically defined substances were confronted with tumour-bearers' leucocytes (Black *et al.*, 1976; Kadish *et al.*, 1976; McCoy *et al.*, 1978). However, even in this condition no unequivocal identification of the relevant antigen was achieved.

Our approach to the identification of the relevant antigens was determined by a relatively common result emerging from LMT studies in different tumour systems, namely that the specificity of the diagnostic reaction is related to the organ of tumour origin (*i.e.* leucocytes from patients with a given tumour "reacted" significantly more frequently with extracts from tumours [autologous as well as allogeneic] arising in the same organ than with extracts from tumours of another organ). Leucocytes from patients with tumours differing in histology (and/or histogenesis) but arising in the very same organ showed no segregation into histologically defined reaction classes (McCoy *et al.*,

This paper is dedicated to Prof. Dr K. E. Scheer, director of the Institute of Nuclear Medicine, German Cancer Research Centre, on the occasion of his 60th birthday.

* Present address: Department of Immunology, Biomedical Centre, University of Uppsala, Uppsala, Sweden.

Address for reprints: Dr S. Matsku, Institute of Nuclear Medicine, German Cancer Research Centre, Im Neuenheimer Feld 280, D-6900 Heidelberg.

1977; Zöller *et al.*, 1980). Since cross-reactivity in tumour immunology is often attributed to the re-expression of foetal antigens, extracts from whole human foetuses (WFE) were examined by LMT. It was determined that WFE, but not extracts from normal adult tissues, gave reactivity with tumour patients' leucocytes, whereas leucocytes from patients with benign disease were essentially unreactive (Zöller *et al.*, 1979). The fact that leucocytes from patients with widely differing tumours nevertheless reacted with a single WFE preparation (although only in 55–60% of cases) seemed to indicate that organ-related foetal specificities will certainly be contained in the WFE, but only in very low concentrations. Thus, leucocytes from patients with different tumours may have reacted with the corresponding organ-related antigens contained in WFE. This hypothesis was tested in the present study. Extracts from specific foetal organs (OFE) were tested with leucocytes from patients with corresponding organ-related and organ-unrelated tumours; other appropriate controls were also included.

### MATERIAL AND METHODS

*Patients.*—Blood samples, tumours and normal tissue samples were provided by physicians affiliated to the Department of Surgery, University of Heidelberg, and the Krankenhaus Rohrbach, Heidelberg*. Tumour patients were tested pre-therapeutically (*i.e.* usually before surgical removal of the primary tumour). No discrimination between different stages was attempted. Data were only included in the final analysis, when tumour diagnosis was confirmed by histology. Blood samples from healthy donors were provided by the local blood bank. Age and sex distribution of leucocyte donors are outlined in the footnote to Table II. Human foetuses were obtained from therapeutic abortion at the City Hospital, Nottingham. Foetal organs were carefully dissected and extracted immediately thereafter.

*Extracts.*—Tissues were finely minced with scissors and extracted using a modification (Zöller *et al.*, 1977*a*) of the 3M KCl method (Reisfeld & Kahan, 1970). Extracts sterilized by filtration were adjusted to protein concentrations of 5, 3 and 1 mg/ml and stored at −20°C. Extracts were prepared from 5 adenocarcinomas of the stomach, 5 adenocarcinomas of the colon or rectum, 5 oat-cell carcinomas of the lung, 5 hypernephromas and from an individual specimen of non-tumorous ("normal") gastric mucosa, colonic mucosa, lung and skin. The gestational age of foetuses as estimated by measurement of the length from crown to rump was found to be around the 12th week. One foetus (length 7·5 cm) was extracted as a whole. Six of the foetuses (length 7·5–12 cm) were carefully dissected into the following organs: skin, brain, lung, gut (excluding stomach), liver, and kidneys. Organs from 3 foetuses were pooled and extracted as outlined above. We thus obtained 2 batches of extract per organ, which gave comparable results in the test. Hence, results obtained with both batches were pooled.

*Blood samples.*—Venous blood was collected into syringes containing 75 u heparin/ml (Kettelhack, Minden, FRG) and processed within 2–3 h of collection.

*Direct capillary tube test.*—The preparation of leucocytes and the direct capillary-tube procedure closely followed the description given previously (Zöller *et al.*, 1979). Each patient's leucocytes were tested only once, by using a set of extracts consisting of 3 tumour extracts, 4 OFE, 1 WFE, and—in some tests—one normal tissue extract. Tumour and normal tissue extracts were tested at a protein concentration of 5 and 1 mg/ml, foetal extracts were tested at 3 and 1 mg/ml.

*Test evaluation.*—Migration plates were photographed and the negatives were projected on to the screen of an AMO2 graphic analyser (Kontron GmbH, München, FRG), which produced digital area equivalents in arbitrary units. The migration index (MI) was calculated from the formula

$$\mathrm{MI} = \frac{\text{mean migration area of test samples}}{\text{mean migration area of control samples}}$$

It has to be mentioned that our test procedure, which is characterized by high extract

* We are indebted to Prof. H. Penzholz and Drs U. Schulz, D. Zeidler and P. Drings for providing the samples and for giving access to patients' data.

TABLE I.—*Leucocyte migration reactivity of cancer patients' leucocytes with corresponding and non-corresponding tumour extracts. Summary of data obtained in diagnostic studies centred around tumours of different organs (references in text)*

| Leucocyte donor (tumour localization) | Positive reactivity* with a panel of tumour extracts from | | | |
|---|---|---|---|---|
| | Stomach (%) | Colon (%) | Lung (%) | Kidney (%) |
| Stomach | 66/77† (86) | 2/10 (20) | 6/18 (33) | |
| Colorectum | 40/63 (63) | 55/59 (93) | 3/10 (30) | |
| Other GI local | 5/18 (28) | 3/23 (13) | 1/5 (20) | |
| Lung | 12/32 (38) | 2/34 (6) | 265/372 (71) | |
| Kidney | 2/9 (22) | 4/19 (21) | 5/30 (17) | 27/37 (73)‡ |
| Breast | 2/25 (8) | 0/15 (0) | 5/24 (21) | |
| Other local. | 30/97 (31) | 2/78 (3) | 47/125 (38) | |

* Significant MI with 3/5 tumour extracts.
† Positive reactions per total number of patients tested.
‡ 28 of these patients are included in Table II.

concentrations and short incubation (Zöller *et al.*, 1977*a*) produced a considerable incidence of migration enhancement. According to previous dose–response experiments (Zöller *et al.*, 1977*a*, 1979) enhancement may be attributed to low antigen concentrations or low lymphocyte sensitization levels or both. In the present context, MIs were classified as "significant" irrespective of whether there was enhancement or inhibition, significance being defined as follows: mean migration areas of test samples (4 replicates) had to differ significantly from the mean migration area of medium samples (12 replicates, Mann–Whitney $U$ test, $P \leqslant 0{\cdot}05$); the response of the 2 extract concentrations had to accord with predetermined dose–response relations; MIs had to be $< 0{\cdot}8$ or $> 1{\cdot}2$. The significance of differences in reaction frequency between different groups of patients was ascertained by the $\chi^2$-test.

### RESULTS

The organ-related reactivity of cancer-patients' leucocytes against soluble tumour extracts is demonstrated by summarizing data collected in the course of studies concerned with gastric (Zöller *et al.*, 1977*a*), colorectal (Zöller *et al.*, 1977*b*) and lung cancer (Zöller *et al.*, 1980). The difference in reaction frequencies with corresponding tumour extracts (71–93% of cases) and with non-corresponding extracts (0–38% of cases) is evident and was shown to be highly significant. These findings, summarized in Table I, together with the demonstration that 55–58% of tumour patients showed significant MIs with extracts from whole human foetuses of gestational ages 10–22 weeks (Zöller *et al.*, 1979) formed the basis of the present study. To test the hypothesis that organ-related foetal determinants may represent the repertoire of antigens responsible for LMT reactivity of tumour-patients' leucocytes, a new series of patients was examined. Leucocytes were tested by 2 different procedures:

(a) Leucocytes from patients with tumours of the lung, stomach, colorectum, kidney and testes were exposed, according to availability, to the whole complement of extracts, including corresponding OFE, non-corresponding OFE, WFE, a panel of 3 tumour extracts and normal tissue extracts. Since amounts of various extracts were limited, the total number of patients tested (far left column in Table II) occasionally differed from the number of patients tested with a given extract.

(b) Leucocytes from patients with other tumours, from patients with benign diseases and from blood-bank donors were tested with foetal extracts only (Table III): this was considered to be an appropriate specificity control at the level of OFE. Extensive specificity-control experiments with tumour extracts and normal tissue extracts have been previously reported (Zöller *et al.*, 1977*a*,*b*, 1979, 1980).

TABLE II.—*Comparison of LMT reactivity induced by foetal organ extracts (OFE), a whole foetal extract, corresponding tumour extracts, and normal tissue extracts*

| Leucocyte donor* (tumour localization) | n | Significant MIs‡ with extracts from: Foetus: Skin | Brain | Lung | Gut | Liver | Kidney | WFE | Corresp. tumours† | Corresp. normal tissue | P§ |
|---|---|---|---|---|---|---|---|---|---|---|---|
| Lung | 11 | — | 0/11 | 6/11 | — | 1/2 | — | — | 6/11 | 0/2 | < 0·01§ |
| Colorectum | 29 | 2/18 | 8/19 | 3/13 | 18/25 | 26/29 | 1/4 | 18/29 | 23/29 | 4/29 | < 0·001 |
| Kidney | 41 | 6/41 | 11/41 | 4/41 | 0/12 | 19/41 | 11/16 | 24/41 | 19/28 | — | < 0·001 |
| Brain | 18 | 0/18 | 11/18 | 2/18 | — | 7/18 | — | 11/18 | — | — | < 0·001 |
| Stomach | 22 | 3/14 | 5/22 | 2/14 | 3/14 | 9/21 | — | 10/22 | 13/17 | 6/17 | n.s. |
| Testis | 10 | 1/10 | 1/10 | 3/10 | 2/4 | 6/10 | 1/4 | 7/10 | 5/10 | 2/10 | n.s. |

* Characteristics of patients: cancer (excluding testicular cancer): 49 F, 72 M; mean age, 61·1 yrs, range 29–88. Testicular cancer: mean age 28 yrs, range 22–33.
‡ Significant MIs per total number of patients.
† Positive reactions per total number of patients tested (see Table I).
§ $\chi^2$ test for the comparison of the reaction frequency with OFE of the corresponding organ *vs* the sum of the frequencies with OFE from non-corresponding organs (excluding liver).

TABLE III.—*Specificity control of LMT reactivity using different foetal extracts*

| Leucocyte donors* (disease groups) | n | Significant MIs with foetal extracts dervied from: Skin | Brain | Lung | Gut | Liver | Whole foetus |
|---|---|---|---|---|---|---|---|
| Cancer patients: | | | | | | | |
| Melanoma | 2 | 2/2† | 0/2 | 0/2 | 1/2 | 1/2 | 1/2 |
| Bone | 3 | 1/3 | 1/3 | 0/3 | — | 1/3 | 3/3 |
| Breast | 9 | 7/8 | 2/9 | 3/9 | 0/8 | 2/8 | 3/9 |
| Oesophagus | 2 | 2/2 | 0/2 | 1/2 | 0/2 | 1/2 | 1/2 |
| Liver | 2 | 0/2 | 0/2 | 0/2 | 0/2 | 2/2 | 2/2 |
| Pancreas | 4 | 1/4 | 1/4 | 0/4 | 0/4 | 1/4 | 3/4 |
| Thyroid | 4 | 1/4 | 0/4 | 0/4 | 0/4 | 2/4 | 2/4 |
| Miscellaneous‡ | 4 | 1/4 | 2/4 | 1/4 | 1/4 | 1/4 | 4/4 |
| Benign disease | | | | | | | |
| Nervous system | 3 | 0/3 | 0/3 | 0/3 | — | 1/3 | 0/3 |
| Gastrointest. system | 11 | 0/11 | 1/11 | 1/11 | 2/8 | 2/11 | 0/11 |
| Respiratory tract | 10 | 0/3 | 1/10 | 1/10 | — | 0/3 | 2/10 |
| Urogenital system | 10 | 0/10 | 0/10 | 1/10 | — | 0/10 | 1/10 |
| Blood-bank donors | 36 | 1/25 | 1/26 | 3/25 | 4/36 | 5/26 | 2/22 |

* Patients' characteristics: Cancer patients, 16 F, 14 M; mean age 56·4 yrs, range 29–94. Benign disease, 12 F, 22 M; mean age 41·3 yrs, range 17–76. Blood-bank donors, 15 F, 21 M; mean age 27·5 yrs, range 22–43.
† Significant MIs per total number of patients tested.
‡ 2 tumours of the pineal gland, 1 tumour of the gallbladder and 1 laryngeal tumour.

The following observations are of significance: OFE-induced reactivity was predominantly obtained with leucocytes from patients with tumours of the corresponding organ. This was reflected by a significant $\chi^2$ test when reaction frequencies obtained from different OFEs (excluding foetal liver extract) were compared. Both batches of foetal liver extracts showed a high percentage of significant MIs with leucocytes from most groups of patients. Wherever OFE, WFE and the tumour extracts were tested simultaneously (*i.e.* with leucocytes from patients with tumours of the colorectum and the kidneys), the highest frequencies of significant MIs were regularly observed with corresponding OFE, though differences from WFE and corresponding tumour extracts were not significant. It may well be that even higher scores could be achieved by using OFE at a protein concentration of 5 mg/ml, which was routine with the other types of extract.

Under the present conditions it was obvious that some of the tumour patients failed to show significant MIs, even when tested with corresponding OFE or tumour extracts. This of course limits the applicability of OFE as a test reagent in clinical diagnosis.

A surprising observation was made with leucocytes from breast-cancer donors (Table III) in that they showed pronounced reactivity with foetal skin extracts. No other groups displaying a high reaction frequency were observed in tests with other types of tumour, which may be due to the paucity of donors in individual tumour groups.

No additional information as to extent and specificity of reactivity was obtained when "significant" MIs were classified according to inhibition or enhancement of migration. With the groups of tumour patients listed in Table II and in the upper part of Table III, 0–24% of significant MIs were found to be > 1·2; with leucocytes from patients with benign disease and from blood-bank donors, enhancement amounted to 7–32% of significant MIs. However, this trend toward a higher proportion of enhancement with control donors' leucocytes could not be substantiated by statistical analysis, mostly because the absolute incidence of enhancement in individual groups was rather small. It should be added that both batches of foetal liver extracts rarely showed enhancement (*i.e.* in 5 and 11% of tests with significant MIs). Hence, the exceptional results with foetal liver extracts could not be explained by exceptionally high incidence of enhancement.

The data from Tables II and III were summarized in order to reveal organ-related and non-organ-related reactivities most readily. As seen in Table IV, highest reaction frequencies were obtained with corresponding OFEs, but reaction frequencies obtained with tumour extracts and WFE were comparable. Reaction frequencies obtained with non-corresponding OFE were definitely lower, thus reinforcing the proposal that sensitization of leucocytes is against antigens of a restricted specificity. Tests with leucocytes from patients with benign disease and with blood-bank donors showed low frequencies of significant MIs (see Table III) and are not included in this summary.

### DISCUSSION

Evidence from LMT analyses of human tumours argues for organ-related antigens triggering the immune response of the host. This concept is strongly supported by the present study, since we were able to show that extracts from foetal tissues preferentially induce organ-related reactivity in patients' leucocytes. Similar results were obtained previously. Wells *et al.* (1973) observed delayed-type hypersensitivity reactions of lung-cancer patients on injection of foetal lung extract (16 weeks gestational age), while no reaction was observed with foetal liver

TABLE IV.—*Organ-related* vs *non-organ-related reactivity: summary af Tables II and III*

| Leucocyte donor* (tumour localization) | n | Significant MIs obtained with extracts from: Corresp. foetal organ (%) | Corresp. tumour (%) | Non-corresp. foetal organ (%) | Whole foetus (%) |
|---|---|---|---|---|---|
| Brain | 18 | 11/18† (61) | — | 2/36‡ (6) | 11/18† (61) |
| Lung | 11 | 6/11 (55) | 24/55‡ (44) | 0/11 (0) | 4/11 (36) |
| Colorectum | 29 | 18/25 (72) | 94/145 (65) | 14/64 (22) | 18/29 (62) |
| Kidney | 41 | 11/16 (69) | 81/140 (58) | 21/135 (16) | 24/41 (59) |

* Individual patients were tested with one corresponding OFE, up to 3 non-corresponding OFE, 1 WFE and 5 tumour extracts.

† Number of significant MIs per total number of patients.

‡ Number of significant MIs per total number of tests (individual patients being tested with more than one tumour extract).

extract. Levin *et al.* (1975) reported a blastogenic response of leucocytes from patients with ovarian cancer toward extracts from foetal and adult ovary, but not to foetal liver nor lung extracts.

Corroboration of the notion of organ-related reaction specificity by tumour-patients' leucocytes in the LMT is the major issue of the present work, using extracts from isolated foetal organs. In addition, the use of OFE helped us to exclude artefacts inherent in the method. It could for instance be possible that organ-specific reactivity of colorectal-cancer extracts were due to microbial contaminants—despite the care we took to keep our extracts sterile. Since an *a priori* microbial contamination of foetal gut preparations is unlikely, data obtained with these extracts help to exclude interferences of this kind. With renal-cancer extracts, it may be argued that immune complexes could be present in tumorous and non-tumorous parts of the tissues used for extraction. We have indeed been able to show that preformed immune complexes can induce strong migration inhibition under appropriate assay conditions (S. Matzku, unpublished). Again, this possibility is rather unlikely with foetal kidney extracts.

However, there is still another possible cause of organ-related reactivity in the LMT as applied to cancer patients' leucocytes. It has been demonstrated in rodents (Martin & Martin, 1975; Pierotti & Colnaghi, 1976) and humans (Morton, 1971; Bloom, 1972; Rosenberg, 1977) that sera from normal or tumour-bearing individuals often contain natural antibodies. By absorption experiments it was demonstrated that these may show organ-related specificities (Martin & Martin, 1975). Hence it could be speculated that specificity in the LMT may be generated by natural antibodies endogenously produced in the test, which could bind to organ-related antigens and ultimately lead to inhibition or enhancement of migration (see above). This interpretation would also apply to tests using foetal extracts. On the basis of the present data it is not possible to discriminate between antibody-mediated reaction mechanisms and the conventional interpretation of the LMT as an *in vitro* analogon of the delayed type hypersensitivity reaction.

In our study foetal liver extracts may be an exception, in as much as both batches induced a high frequency of significant migration indices (mostly inhibition) with leucocytes from tumour patients but also (to a lesser extent) with leucocytes from patients with benign diseases and from blood-bank donors. The microscopic aspect of leucocyte halos after exposure to foetal liver extracts often suggested that these extracts were in fact unspecifically cytotoxic. Yet it is noteworthy that among the different groups, leucocytes from patients with colorectal cancer showed an exceedingly high score of significant MIs. In this groups it may well be that "specific" reactivity and unspecific cytotoxicity by foetal liver extracts were superimposed.

When OFEs were tested with leucocytes from either patients with tumours originating in non-corresponding organs or patients with benign diseases or blood-bank donors, we observed significant MIs in only 0–22% of tests. This contrasted with the high frequency of significant MIs on interaction of OFE with leucocytes from patients with tumours in the corresponding organs. Hence, it can be postulated that the reactivity induced by foetal organ extracts is attributable to their content of substances specifically interacting with sensitized lymphocytes. The background of reactivity in tests with non-corresponding leucocytes, or leucocytes from patients devoid of malignant disease, may be caused either by antigens with broad specificity or by artefactual interactions between leucocytes and extracts. However, it has to be pointed out that an in-depth analysis of LMT reactivity by leucocytes from patients with selected benign diseases possibly involving autoimmune phenomena may reveal specific sensitization to substances also contained in organ-related foetal extracts. This was indeed observed by Marcussen & Bendixen (1974) when leucocytes from patients with ulcerative colitis were confronted with an extract of foetal colon—but not with an extract of foetal lung.

In the present study, we have used pools of foetal tissue for extraction. However, the gestational age of foetuses varied only between 10 and 12 weeks. This may explain the lack of OFE-induced reactivity with some patients. It is possible that tumours of different histological types, and even individual tumours with similar histology, may carry antigens which are expressed during different phases of gestation. Interpretations of this kind have been proposed by various authors (for references see Rees *et al.*, 1979) but logistic and legal difficulties obviously prevented experimental verification. In the mouse, evidence for phase-specific foetal determinants being expressed on tumour cells was obtained (Gorczynsky, 1978).

There may be other interpretations for the occasional lack of reactivity of cancer patients' leucocytes with corresponding OFEs. Since migration enhancement appears to be produced by a distinct lymphokine (*i.e.* the migration-enhancement factor of Weisbart *et al.* (1974)) it is conceivable that under certain conditions liberation of inhibition factor and enhancement factor may occur simultaneously, and the counteracting effects might be balanced. Obviously, this situation would be misclassified as absence of sensitization. Furthermore, absence of *in vitro* reactivity might reflect a subthreshold level of sensitization *in vivo*, which might either be caused by negative regulation or by an insufficient expression of antigens on the tumour cells. Finally, technical problems may account for a lack of reactivity (*e.g.* insufficient test sensitivity, instability of foetal antigens under extraction conditions).

Two conclusions can be drawn from the above arguments. First, widespread reactivity of WFE in immunodiagnostic tests such as the LMT (Albrecht *et al.*, 1978; Zöller *et al.*, 1979) is not at variance with organ-related reaction specificity of tumour extracts. According to our initial hypothesis, widespread cross-reactivity could be dissected into more or less unidirectional components by separate extraction of foetal organs. Second, in contrast to our expectation, no qualitative and only slight quantitative improvement of LMT sensitivity could be achieved by the use of OFE. Hence, even with the use of OFE, it cannot be expected that tumour diagnosis by the LMT will permit prognoses with validity for the individual patient. Yet OFE might be a useful starting material for the preparation of oncofoetal antigens.

### REFERENCES

ALBRECHT, S., LE THANH THUY, PASTERNAK, G. & 5 others (1978) Leukozytenmigrations-Inhibitions (LMI) und Leukozyten-Adharenz-Inhibitions (LAI) Test bei Patienten mit Tumoren unter Verwendung von Fetalantigen. *Acta Biol. Med. Germ.*, **37**, 1747.

BLACK, M. M., ZACHRAU, R. E., DION, A. S. & 4 others (1976) Cellular hypersensitivity to gp55 of RIII-murine mammary tumor virus and gp55-like protein of human breast cancers. *Cancer Res.*, **36**, 4137.

BLOOM, E. T. (1972) Further definition by cytotoxicity tests of cell surface antigens of human sarcomas in culture. *Cancer Res.*, **32**, 960.

GORCZYNSKY, R. M. (1978) Response of tumour-related and normal lymphocytes to antigens on fibroblasts from embryos of varying age. *Br. J. Cancer*, **37**, 786.

KADISH, A. S., MARCUS, D. M. & BLOOM, B. R. (1976) Inhibition of leukocyte migration by human breast cancer-associated antigens. *Int. J. Cancer*, **18**, 581.

LEVIN, L., MCHARDY, J. E., CURLING, O. M. & HUDSON, C. N. (1975) Tumour antigenicity in ovarian cancer. *Br. J. Cancer*, **32**, 152.

MARCUSSEN, H. & BENDIXEN, G. (1974) Circulating antibodies and leukocyte migration inhibition to colon-associated antigen in ulcerative colitis and Crohn's disease. *Scand. J. Gastroenterol.*, **9**, 149.

MARTIN, S. W. & MARTIN, W. J. (1975) Anti-tumour antibodies in normal mouse sera. *Int. J. Cancer*, **15**, 658.

MCCOY, J. L., JEROME, L. F., CANNON, G. B., WEESE, J. L. & HERBERMAN, R. B. (1977) Reactivity of lung cancer patients in leukocyte migration inhibition assays to 3M potassium chloride extracts of fresh tumor and tissue cultured cells derived from lung cancer. *J. Natl Cancer Inst.*, **59**, 1413.

MCCOY, J. L., DEAN, J. H., CANNON, G. B. & 5 others (1978) Leukocyte migration inhibition and lymphocyte blastogenesis responses in breast carcinoma patients to mouse mammary tumor virus and to virion gp52 antigen and Rauscher Murine

Leukemia Virus–Kirsten Sarcoma Virus gp69/71 antigen. *J. Natl Cancer Inst.*, **60**, 1259.

MORTON, D. L. (1971) Immunological studies with human neoplasms. *J. Reticuloendothel. Soc.*, **10**, 137.

PIEROTTI, M. A. & COLNAGHI, M. I. (1976) Natural antibodies against murine lymphosarcoma cells: Variability of levels in individual mice. *Int. J. Cancer*, **18**, 223.

REES, R. C., PRICE, M. R. & BALDWIN, R. W. (1979) Oncodevelopmental antigen expression in chemical carcinogenesis. *Methods Cancer Res.*, **18**, 99.

REISFELD, R. A. & KAHAN, B. D. (1970) Biological and chemical characterization of human histocompatibility antigens. *Fed. Proc.*, **29**, 2034.

ROSENBERG, S. (1977) Lysis of human normal and sarcoma cells in tissue culture by normal human serum. Implications for experiments in human immunology. *J. Natl Cancer Inst.*, **58**, 1233.

WELLS, S. A., BURDICK, J. F., CHRISTIANSEN, C., KETCHAM, A. S. & ADKINS, P. C. (1973) Demonstration of tumor associated delayed cutaneous hypersensitivity reactions in patients with lung cancer and in patients with carcinoma of the cervix. *Natl Cancer Inst. Monogr.*, **37**, 197.

WEISBART, R. H., BLUESTONE, R., GOLDBERG, L. S. & PEARSON, C. M. (1974) Migration enhancement factor: A new lymphokine. *Proc. Natl Acad. Sci. U.S.A.*, **71**, 875.

ZÖLLER, M., MATZKU, S. & SCHULZ, U. (1977*a*) Leukocyte migration studies in gastric cancer detection: An approach toward improved specificity and sensitivity. *J. Natl Cancer Inst.*, **58**, 897.

ZÖLLER, M., MATZKU, S. & SCHULZ, U. (1977*b*) Colorectal cancer diagnosis by a direct leukocyte migration test using a panel of tumour extracts. *Cancer Immunol. Immunother.*, **2**, 257.

ZÖLLER, M., MATZKU, S., SCHULZ, U. & PRICE, M. R. (1979) Sensitization of leukocytes of cancer patients against fetal antigens: Leukocyte migration studies. *J. Natl Cancer Inst.*, **63**, 285.

ZÖLLER, M., MATZKU, S. & ZEIDLER, D. (1980) Crossreactivity of lung cancer patients' leukocytes in the leukocyte migration inhibition test. *Cancer Immunol. Immunother.*, **8**, 143.